# Hypertensive crisis in children: an experience in a single tertiary care center in Korea

**DOI:** 10.1186/s40885-016-0040-2

**Published:** 2016-04-18

**Authors:** Geum Hwa Lee, I Re Lee, Se Jin Park, Ji Hong Kim, Ji Young Oh, Jae Il Shin

**Affiliations:** 1 Department of Pediatrics, Yonsei University College of Medicine, Seoul, Korea; 2 Department of Pediatrics, Daewoo General Hospital, Ajou University School of Medicine, Geoje, Korea; 3 Department of Pediatric Nephrology, Severance Children’s Hospital, Seoul, Korea; 4 Department of Pediatrics, Gangnam Severance Hospital, Yonsei University College of Medicine, Seoul, Korea

**Keywords:** Hypertensive crisis, Antihypertensive drugs, Children

## Abstract

**Background:**

Hypertensive crisis is a medical emergency that can cause acute damage to multiple end-organs. However, relatively little is known on the etiology, treatment, and outcomes of hypertensive crisis in Korean children. The aim of this study was to determine the etiologies and efficacy of drugs for hypertensive crisis in children during the past 5 years at a single center in Korea.

**Methods:**

We analyzed data from 51 children with hypertensive crisis during the period between January 1, 2010 and April 1, 2014. The patients were divided into two groups: those diagnosed with a hypertensive emergency (hypertension with organ injury, n = 31) and those diagnosed with a hypertensive urgency (hypertension without organ injury, n = 20). Baseline etiologies and risk factors were compared between the two groups. In addition, systolic and diastolic blood pressures were evaluated at 1, 2, 4, and 5 hours after the administration of intravenous antihypertensive drugs.

**Results:**

Kidney injury and cancer were the common causes in patients with hypertensive crisis. Cardiovascular complications (cardiac hypertrophy) (p = 0.002), central nervous system complications (p = 0.004), and retinopathy (p = 0.034) were more frequently observed in children with hypertensive emergency than those with hypertensive urgency. However, the proportion of renal complications was similar in both groups. Hydralazine was most commonly used in both groups to control acute increasing blood pressure at first. However, it was often ineffective for controlling abrupt elevated blood pressure. Therefore, intravenous antihypertensive drugs were changed from hydralazine to nicardipine, labetalol, or nitroprusside to control the high blood pressure in 45.1 % of the patients. Particularly, in patients with hypertensive crisis, there was no significant difference in reduction of systolic and diastolic blood pressure and in improvement of clinical outcomes between nicardipine and labetalol administration.

**Conclusion:**

Close blood pressure monitoring and careful examinations should be mandatory in children with underlying disease, especially renal diseases and cancer. Furthermore, both nicardipine and labetalol may be effective antihypertensive drug in lowering high blood pressure in children with hypertensive crisis.

## Background

Hypertension in children is defined as a sustained systolic blood pressure (SBP) or diastolic blood pressure (DBP) elevation status greater than or equal to the 95th percentile for age, gender, and height [1]. Several studies including National Health and Nutrition Examination Survey (NHANES) have shown that the morbidity of hypertension in children and adolescents is about 1-4 %, and the average blood pressure is recently increasing during childhood [1–3].

Hypertensive crisis is a clinical syndrome of severe hypertension that can cause life-threatening status [4–6]. This term can be subdivided into two groups: a “hypertensive emergency,” which has signs of organ failure, and a “hypertensive urgency,” which does not have any other complication except elevation of blood pressure [7–11]. Approximately 20 % to 40 % of hypertensive crises are hypertensive emergencies, and 60 % to 80 % are hypertensive urgencies [11, 12]. Hypertensive emergency can lead to multiorgan damage, requiring urgent management to reduce blood pressure [4–6]. The organs susceptible to impairment include the brain, eyes, heart, and kidneys, with the major pathological process being fibroid necrosis of arterioles [6].

Hypertensive crisis, however, is rare in children, and the prevalence of hypertensive crisis in children is currently unknown [13]. In addition, studies on the choice for the initial antihypertensive drugs are still lacking. The aim of this study was to determine the etiologies and efficacy of drugs for hypertensive crisis in children.

## Methods

The medical records were retrospectively reviewed to search for patients with hypertensive crisis admitted to Yonsei University Severance Hospital during the study period (January 1, 2010 to April 1, 2014). The inclusion criteria for patients were as follows: 1) below 20 years of age; 2) diagnosis of hypertensive crisis which has symptoms and significant elevations in blood pressure with or without accompanying end-organ damage; and 3) treated with intravenous hydralazine or continuous nicardipine, labetalol, and nitroprusside infusion. Participants were excluded if they had an immediately-postoperative status. Therefore, we enrolled a total number of 51 patients.

The patients with hypertensive crisis were divided into two groups according to the presence of end-organ damage: hypertensive emergency (organ injury, n = 31) and hypertensive urgency (no organ injury, n = 20). The definition of end-organ damage was having signs or symptoms suggesting a hypertensive emergency such as visual symptoms, seizure, anuria, hematuria, or abnormal findings in a 12-lead electrocardiogram or echocardiography. Data collected included age, sex, and etiology of hypertension. Additionally, serum blood urea nitrogen (BUN) and creatinine (Cr) were collected as laboratory data. SBP and DBP were measured hourly after continuous infusion of antihypertensive agent. Baseline etiologies and characteristics were also compared between the two groups.

Baseline blood pressure was also assessed and was defined as the patient's highest blood pressure before starting the medication. Blood pressure was then measured every hour until 5 hours after initiation of the antihypertensive medication.

For statistical analysis, continuous-value data for each group were compared using a student *t*-test, and a chi square test was used for categorical variables using SPSS version 18.0 (SPSS Inc., Chicago, IL, USA). A *p*-value of <0.05 was considered to have statistical significance. This study was approved by the Institutional Review Board (IRB) of Severance Hospital (Seoul, Korea, IRB No. 4-2015-0144).

## Results

Baseline etiologies were presented in Table 1. Cancer was the most common cause (47.0 %) of hypertensive crisis in children. Most of cancer patients showed hypertensive crisis after receiving chemotherapy such as cyclophosphamide, cisplatin and methotrexate, known as nephrotoxic agents. Only 3 cases of Wilms’ tumor showed hypertensive crisis by cancer mass itself. Kidney injury was the second common cause (29.5 %), which contained three subtype etiologies: renal disease (15.7 % of the patients), post-renal disease (6.0 %), and renal artery stenosis (7.8 %). Particularly, patients with renal diseases consisted of acute poststreptococcal glomerulonephritis (APSGN), thrombotic microangiopathy (TMA), and drug-induced tubulointerstitial nephritis (TIN). Baseline characteristics were similar between the two groups with respect to age, sex, and etiologies. Only hypoxic brain injury patients were higher in the hypertensive urgency group than in the hypertensive emergency group (p = 0.029) (Table 2).

**Table 1:** Etiologies of the patients who were treated for hypertensive crisis

Etiology	Number of patients	Percentage (%)
Kidney origin	15	29.5
Renal disease	8	15.7
APSGN	3	6.0
Nephrotic syndrome	2	3.9
TMA	2	3.9
Drug-induced TIN	1	2.0
Postrenal disease	3	6.0
Reflux nephropathy	1	2.0
Ureter stricture	1	2.0
Post renal stone	1	2.0
Renal artery stenosis	4	7.8
Cancer	24	47.0
Wilms’ tumor	3	6.0
Neuroblastoma	2	3.9
Other solid tumor	7	13.7
Hematologic cancer	12	23.5
Sepsis	4	7.8
Hypoxic brain injury	6	11.8
Cardiogenic	2	3.9
Total	51	100

*APSGN*, acute poststreptococcal glomerulonephritis; *TMA*, thrombotic microangiopathy; *TIN*, tubulointerstitial nephritis

**Table 2:** Comparison of basal characteristics of the patients with hypertensive emergency and urgency

Characteristics	Hypertensive emergency (N = 31)	Hypertensive urgency (N = 20)	*P* value
Age (year)	8.46 ± 5.20	5.56 ± 4.71	0.051
Sex			
Male (%)	18 (58.1)	10 (50.0)	0.572
Female (%)	13 (41.9)	10 (50.0)	
Etiology			
Renal origin (%)	10 (32.3)	5 (25.0)	0.951
Renal disease (%)	5 (16.1)	3 (15.0)	1.000
Postrenal disease (%)	2 (6.4)	1 (5.0)	1.000
Renal artery stenosis (%)	3 (9.8)	1 (5.0)	1.000
Cancer (%)	16 (51.7)	8 (40.0)	0.267
Sepsis (%)	2 (6.4)	2 (10.0)	0.640
Hypoxic brain injury (%)	1 (3.2)	5 (25.0)	0.029
Cardiogenic (%)	2 (6.4)	0 (0.0)	0.514

Systemic signs and symptoms suggestive of potential end-organ damage were presented in Table 3. Hypertensive emergencies caused the target organ damage to the ophthalmologic, neurologic, cardiologic, and nephrologic systems. Visual symptoms between the two groups did not show the significant difference, but retinopathy was more frequently observed in the hypertensive emergency group than in urgency group (p = 0.034) on ophthalmologic examination. Because two patients’ ophthalmologic examination results were normal, they were classified as hypertensive urgency group despite of having eye symptoms. In the hypertensive emergency group, 29.0 % of the patients had seizures, and 62.5 % of the patients were diagnosed with posterior reversible encephalopathy syndrome (PRES), whereas no patients had either sign (p = 0.004, p = 0.004, respectively) in the hypertensive urgency group. Abnormal cardiovascular signs such as cardiac hypertrophy were found in 43.3 % of the patients on baseline 12-lead electrocardiogram and in 40.9 % of the patients on echocardiogram in the hypertensive emergency group, whereas no patient had such signs in the hypertensive urgency group, with significant differences (p = 0.002, p = 0.002, respectively). Kidney-related symptoms and signs, including anuria, creatinine elevation, BUN/Cr level, hematuria, and proteinuria, did not show significant difference except for renal ultrasonography, in which the hypertensive emergency group showed more abnormal results (p = 0.025).

**Table 3:** Target organ damage of various organs in patients with hypertensive crisis

	Hypertensive emergency (N = 31)	Hypertensive urgency (N = 20)	*P* value
EYE			
Visual symptom (%)	7 (22.6)	2 (10.0)	0.454
Retinopathy (%)	7/14 (50.0)	0/6 (0.0)	0.034
CNS			
Seizure (%)	10 (29.0)	0 (0.0)	0.004
PRES on brain MRI (%)	10/16 (62.5)	0/11 (0.0)	0.004
HEART			
LVH, RVH, BVH (%)	13/30 (43.3)	0/15 (0.0)	0.002
Abnormal EchoCG (%)	9/22 (40.9)	0/10 (0.0)	0.002
Ejection fraction	68.7 ± 9.70	68.1 ± 5.34	0.434
Kidney			
Anuria (%)	14 (45.2)	8 (40.0)	0.778
Cr elevation (%)	15 (48.4)	9 (45.0)	1.000
BUN	27.07 ± 20.49	23.91 ± 19.80	0.294
Cr	1.08 ± 1.24	0.87 ± 1.13	0.278
Hematuria (%)	12 (38.7)	8 (40.0)	1.000
Proteinuria (%)	7 (22.6)	6 (30.0)	0.466
Abnormal renal USG (%)	10/20 (50.0)	0/8 (0.0)	0.025

*CNS*, central nervous system; *PRES*, posterior reversible encephalopathy syndrome; *LVH*, Left ventricular hypertrophy; *RVH*, right ventricular hypertrophy; *BVH*, biventricular hypertrophy; *EchoCG*, echocardiogram; *Cr*, serum creatinine; *BUN*, blood urea nitrogen; *USG*, ultrasonography

Treatment of the patients with hypertensive crisis included hydralazine, nicardipine, and labetalol (Table 4). Hydralazine was most commonly used to control acute increasing blood pressure for the first time in 26 (51 %) of the 51 patients. However, it was often ineffective for controlling abrupt elevated blood pressure in 23 (88.5 %) of the 26 patients. Therefore, the antihypertensive drug was changed from hydralazine to nicardipine, labetalol, or nitroprusside to control the high blood pressure in the patients. Six labetalol infusions and three nitroprusside infusions were changed into nicardipine due to poor blood pressure control. Only one patient switched from nitroprusside to labetalol, and there were no patients who were transferred from nicardipine to other medications.

**Table 4:** Treatment in patients with hypertensive crisis

Categories	Hypertensive emergency (N = 31)	Hypertensive urgency (N = 20)	Total (N = 51)
HD → NCR (%)	10 (32.3)	3 (15.0)	13 (25.5)
NCR only (%)	4 (12.9)	5 (25.0)	9 (17.6)
HD → LAB (%)	3 (9.7)	4 (20.0)	7 (13.7)
LAB only (%)	5 (16.1)	1 (5.0)	6 (11.8)
HD → NTP (%)	0 (0.0)	3 (15.0)	3 (5.9)
NTP only (%)	0 (0.0)	0 (0.0)	0 (0.0)
Transition (%)	6 (19.4)	4 (20.0)	10 (19.6) ^†^
LAB → NCR (%)	4 (12.9)	2 (10.0)	6 (11.8)
NCR → LAB (%)	0 (0.0)	0 (0.0)	0 (0.0)
NTP → NCR (%)	3 (9.7)	0 (0.0)	3 (5.9)
NTP → LAB (%)	0 (0.0)	1 (5.0)*	1 (2.0)
HD only (%)	3 (9.7)	0 (0.0)	3 (5.9)

*HD*, hydralazine; *LAB*, labetalol; *NCR*, nicardipine; *NTP*, nitroprusside

* 1 patient used NCR → NTP → LAB

^†^Transition time: mean 50 hours

**Fig. 1:**
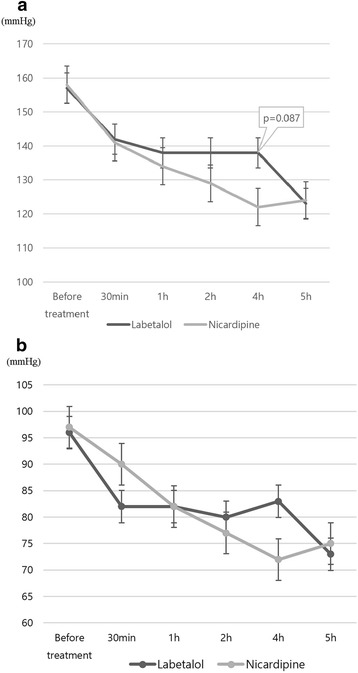
Changes in systolic (**a**) and diastolic (**b**) blood pressure by administration of labetalol and nicardipine during 5 hours

In both hypertensive emergency and hypertensive urgency group, there was no significant difference in reduction of SBP and DBP between nicardipine and labetalol administration (Table 5). However, nicardipine showed more decreased SBP at 4 hours than labetalol, although it had a borderline significance (Fig. 1).

**Table 5:** Comparison of the effect of nicardipine and labetalol on systolic and diastolic blood pressure

	Systolic blood pressure (mmHg)			Diastolic blood pressure (mmHg)		
	Nicardipine	Labetalol	P-value	Nicardipine	Labetalol	P-value
Before treatment	158.00 ± 22.56	157.08 ± 23.86	0.756	97.54 ± 20.69	96.31 ± 14.80	0.812
30 min	141.81 ± 25.02	142.54 ± 141.81	0.651	90.09 ± 18.93	82.54 ± 16.97	0.885
1 h	134.95 ± 19.29	138.38 ± 24.88	0.498	82.27 ± 16.90	82.62 ± 19.31	0.522
2 h	129.41 ± 16.20	138.23 ± 20.81	0.105	77.32 ± 15.39	80.92 ± 18.59	0.433
4 h	122.27 ± 13.59	138.46 ± 22.20	0.087	72.23 ± 18.62	83.31 ± 18.56	0.827
5 h	124.59 ± 17.95	123.18 ± 11.98	0.167	75.77 ± 17.52	73.45 ± 16.81	0.746

We further evaluated the clinical outcomes after finishing treatment of intravenous nicardipine or labetalol single infusion in both hypertensive emergency and urgency patients (Table 6). Total 6 (26.0 %) patients eventually died and 17 (74.0 %) survived in nicardipine group, otherwise 9 (50.0 %) patients died and 9 (50.0 %) survived in labetalol infusion cases. Among these patients, we analyzed again only targeting survivors. In nicardipine group, 10 (58.3 %) patients were taking oral antihypertensive medications after intravenous treatment and 7 (41.7 %) patients were tapered out all medications, whereas 5 (55.6 %) patients were taking oral medications, 4 (44.4 %) patients were not taking any medications in labetalol group. Improvement of visual symptoms, creatinine elevation, hematuria or proteinuria, and abnormality on baseline 12-lead electrocardiogram did not show any significant difference between the two groups.

**Table 6:** Comparison of 1 year follow-up outcomes of the patients who were treated with nicardipine and labetalol

Categories	Nicardipine (N = 23)	Labetalol (N = 18)	P-value
Primary outcomes			
Mortality (%)	6 (26.0)	9 (50.0)	0.191
Survivors (%)	17 (74.0)	9 (50.0)	
Secondary outcomes (in survivors)			
Medication change			
Taking oral medication (%)	10 (58.3)	5 (55.6)	1.000
No medication (%)	7 (41.7)	4 (44.4)	
Clinical improvement			
Visual symptom† (%)	0/2 (0.0)	1/2 (50.0)	1.000
Retinopathy (%)	0/2 (0.0)	1/2 (50.0)	1.000
Seizure (%)	5/5 (100.0)	2/2 (100.0)	-
Cr elevation (%)	7/7 (100.0)	5/6 (83.3)	0.462
Hematuria or Proteinuria (%)	4/8 (50.0)	5/5 (100.0)	0.105
LVH, RVH, BVH on ECG (%)	1/2 (50.0)	2/3 (66.7)	1.000

*Cr*, serum creatinine; *LVH*, Left ventricular hypertrophy; *RVH*, right ventricular hypertrophy; *BVH*, biventricular hypertrophy; *ECG*, electrocardiogram

## Discussion

The main goal of this study was to investigate the causes of pediatric hypertensive crisis and the efficacy of drugs used to control it. We found that cancer and renal disease were the two common causes of hypertensive crisis as described in other studies [14, 15]. Our study also exhibited that there was no difference between nicardipine and labetalol in the treatment of hypertensive crisis, which differed from the result of an adult study that showed nicardipine to be more efficacious within 30 minutes than labetalol in patients with renal dysfunction [16]. However, Thomas et al. also reported no significant difference of efficacy between nicardipine and labetalol in infants and small children with a hypertensive crisis [17]. Particularly, we tried to compare the hypertensive emergency with hypertensive urgency in children.

Although hypertension is usually regarded as a disease of adulthood, with a prevalence of 30 % [18], it can also affect children and adolescents, traditionally with a prevalence of 1 %–2 %. However, recent studies have suggested that it has increased to over 3 %, with a much higher prevalence of 4.5 % in children with obesity [3, 19]. Severe childhood hypertension is associated with adulthood morbidity and mortality as a long-standing elevated blood pressure [14]. Ninety-day mortality rates were reported to be 11 % in patients who were hospitalized and treated in emergency circumstances [20]. These serious situations are related to acute end-organ damage and require immediate, controlled blood pressure reduction, and close observation. Without proper treatment, the 1-year mortality rate of hypertensive emergencies increases to 90 % [10]. However, there is no formal standard of treatment for severely elevated blood pressure in such emergency circumstances in children and adolescents with renal disease. Therefore, this study serves to demonstrate an optimal treatment option for hypertensive crisis patients with renal dysfunction, with results indicating that antihypertensive therapy should be tailored to each patient.

Nicardipine hydrochloride, approved by the Food and Drug Administration in December 1988, belongs to the class of dihydropyridine calcium channel blockers used to treat vascular disorders including high blood pressure, Raynaud’s phenomenon, and chronic stable angina [13], whereas labetalol is a mixed adrenergic antagonist that blocks α1-receptor and nonselective β receptor with an α:β blocking ratio of 1:7 [21]. The action mechanism of nicardipine and its clinical effects closely resemble those of nifedipine and the other dihydropyridines, such as felodipine and amlodipine; however, nicardipine is more selective for cerebral and coronary blood vessels [13]. Moreover, nicardipine does not intrinsically reduce myocardial contractility and has a longer half-life than nifedipine, as labetalol causes a decrease in systemic arterial blood pressure and systemic vascular resistance without a substantial reduction in resting heart rate, cardiac output, or stroke volume, apparently due to its combined α- and β-adrenergic blocking activity [22, 23].

The Seventh Report of the Joint National Committee on Prevention, Detection, Evaluation, and Treatment of High Blood Pressure declared that arterial blood pressure must be decreased by no more than 10 %–25 % during the first hour of treatment [24]. Both nicardipine and labetalol were found to decrease SBP by 14.6 % and 11.9 % within 1 hour in our study, respectively. DBP also decreased to 15.7 % of the initial DBP within 1 hour in nicardipine-treated patients, whereas patients with labetalol treatment had 14.2 % elevated blood pressure within 1 hour. These findings were thought to be generally caused by the fact that appropriate titration, redosing, and monitoring of labetalol were not easy in a busy emergency room, suggesting that more aggressive dosing of labetalol might be required for blood pressure response in patients with hypertensive emergency.

There is concern that iatrogenic effects such as hypotension and bradycardia can occur when using nicardipine and labetalol. Rapid blood pressure declines in nicardipine and labetalol patients were not observed, possibly owing to physician understanding and acknowledgment. In fact, reported adverse events including drowsiness, weakness, hyperkalemia, and drug eruption were uncommon in our study due to the short period of treatment. Both medications are metabolized by the liver; therefore, patients with renal impairment may be treated without profound complications.

We were not able to draw firm conclusions with regard to the comparative efficacy and safety of nicardipine vs. labetalol in children and adolescents with hypertensive crisis. As the data were insufficient for measuring long-term outcomes in patients experiencing hypertensive crises, further research is necessary in the near future. There is a possibility that some with hypertensive emergency may not have been ultimately diagnosed with acute end-organ damage due to a low SBP under 180 mmHg or a DBP under 120 mmHg. In the treatment of such patients without hypertensive emergency, oral antihypertensive medication may have been a reasonable option. It can be very challenging to collect data on children and adolescents with true hypertensive emergencies. Thus, the study population may not represent the patients to whom nicardipine and labetalol would most likely be prescribed. Additionally, ethnic differences between Asian, African, and Caucasian populations should be taken into account.

## Conclusion

In conclusion, there was no difference between nicardipine and labetalol for a rapid and controlled BP decrease of administration in children and adolescents with hypertension and renal disease. However, these results should be considered in the context of the patient population and tempered with further studies to determine whether the controlled blood pressure improvement obtained by nicardipine or labetalol truly has any clinical significance.
